# Sex and the Clock: Time to Consider Sexually Dimorphic Rhythms in Circadian Research

**DOI:** 10.1177/07487304251333548

**Published:** 2025-04-24

**Authors:** Ronan Lordan

**Affiliations:** Institute for Translational Medicine and Therapeutics, Perelman School of Medicine, University of Pennsylvania, Philadelphia, Pennsylvania, USA

The circadian clock is a sophisticated, self-sustained, near-24-hour, internal timing mechanism that anticipates daily environmental cues (i.e. Zeitgebers) to confer temporal structure on behavior and physiology. Growing concern exists that preclinical research predominantly relies on male animals, leading to sex bias that compromises our understanding of female biology and the translation of preclinical findings to humans (Zucker et al., 2022). Sex differences in biological responses to various stimuli are well-documented (Anderson and FitzGerald, 2020). However, the interaction between sex and circadian rhythms is less explored.

Locomotor activity rhythms in rodents are sexually dimorphic and influenced by the estrous cycle (Anderson and FitzGerald, 2020). However, 80% of phase-shifting studies in rodent circadian research over the last 50 years have been performed with exclusively male animals (Lee et al., 2021). Historically, researchers have been hesitant to study the behavior and physiology of female rodents due to concerns about the estrous cycle’s impact on various phenotypes. Recent studies have challenged this reluctance, suggesting that the estrous cycle may not influence female rodent behavior to the magnitude previously thought in many fields of research (Lee et al., 2021). Interestingly, sex hormones are not required to maintain circadian locomotor rhythms, but they do affect the amplitude of the rhythm and the magnitude of the phase-shifting response to photic stimuli (Anderson and FitzGerald, 2020). These interactions add to the complexity of the relationship between sex and the clock.

In a recent issue of the *Journal of Biological Rhythms*, Pastrick et al. (2024) investigated sex differences in locomotor activity and behavioral rhythms in mice under varying environmental conditions. Their studies revealed that male C57BL/6 mice housed in standard light-dark cycles (LD12:12) exhibited periodic rest phases (siestas) in their active phase, measured using wheel running activity. Conversely, the locomotor activity of female mice was significantly higher than that of male mice during the active phase, particularly during proestrus. Female mice also had fewer siestas than male mice, with fewer siestas occurring during the proestrus and estrus stages indicating differences in resting behaviors. In the absence of running wheels, the authors showed that male and female mPer2^Luc^ mice exhibited similar activity and siesta patterns as previously observed, but male mice had more structured and consistent siestas than female mice over the course of the experiment.

To determine how activity rhythms may be affected by long day photoperiods, mPer2^Luc^ mice with or without running wheels were entrained to a 20-hour light photoperiod, a strategy known to induce sexually dimorphic suprachiasmatic nucleus (SCN) rhythms. Compared to LD12:12 conditions, 20-hour light exposure reduced activity rhythms in both sexes, but all mice re-entrained within 2-4 weeks. Notably, male mice without running wheels had a greater percentage of days with a siesta and had symmetrical activity bouts at dawn and dusk. Female mice, however, maintained a consistent break in activity at dawn, regardless of wheel access, suggesting that female mice may be less influenced by housing conditions compared to male mice.

Further experiments exposed mice to LD12:12, LD20:4, or LD4:20 in the presence of running wheels for 10 weeks prior to release into constant darkness (DD). These experiments showed that sex differences in daily wheel running rhythms are modulated by photoperiod length but persist even in DD. Moreover, sexually dimorphic differences in frequency of occurrence of nightly siestas persist in the absence of running wheels under various photoperiod lengths, but male mice show great variability in siesta patterns across all photoperiods compared to female mice. In a separate experiment, the authors also showed that female mice exhibited phase jumps, shifts in activity into the daytime scotophase (dark phase), earlier than male mice when exposed to “skeleton” photoperiods, indicating that parametric responses to gradual changes in daylength differ by sex. Collectively, the photoperiod experiments performed by Pastrick et al. (2024) suggest that male and female mice use different mechanisms to adapt to environmental light cycles.

This study corroborates existing literature that shows that there are sex-specific differences in locomotor activity and circadian rhythms in both C57BL/6 and mPer2^Luc^ mice in response to varying environmental conditions. Collins et al. (2020) showed that nighttime-active VIP+ neurons in the SCN regulate sleep and activity in a gated manner, whereby the clock appears to time siesta occurrence. It is possible that male and female mice have different activity of vasoactive intestinal polypeptide neurons, particularly VIP+SCN neurons, affecting siesta frequency, although other mechanisms are possible. It is worth noting that the behavior of mPer2^Luc^ reporter mice (also on a C57BL/6 background) was characterized in this study, given that they are frequently used in circadian rhythm research; however, PER2::LUC reporter assays were not utilized specifically by Pastrick et al. (2024). In other studies, mPer2^Luc^ mice have demonstrated sexually dimorphic outcomes in locomotor activity, neural activity, and gene expression. Kuljis et al. (2013) showed that male mPer2^Luc^ mice have greater precision in the timing of activity onset than female mice. Moreover, it has been shown that male mice with the PER2::LUC fusion protein may have altered circadian phenotypes in comparison to C57BL/6 mice. Specifically, mPer2^Luc^ mice have a shortened period of circadian locomotor activity rhythms under constant light and a lengthened period of locomotor activity under constant darkness versus C57BL/6 (Ralph et al., 2021). These are phenotypes that were not observed by Pastrick et al. (2024).

This study calls into the question whether sex differences in locomotor activity and light exposure drive sexually dimorphic responses in humans. In particular, it is known that there is a higher prevalence of sleep disorders among women (Kuljis et al., 2013). There is also a robust link between seasonal affective disorder (SAD) and changes in seasonal daylength with up to four times greater prevalence among women (Meesters and Gordijn, 2016). These findings suggest that the circadian clock may be implicated in the higher prevalence of SAD among women. Indeed, entrainers of the clock including activity, exercise, and light exposure are under investigation as therapies for the mitigation of SAD and sleep disorders. Preclinical studies such as that of Pastrick et al. (2024) point to a greater need for understanding of the differences in male and female circadian rhythms and responses to environment cues.
